# Enrichment of Refined Olive Oils with Phenolic Extracts of Olive Leaf and Exhausted Olive Pomace

**DOI:** 10.3390/antiox11020204

**Published:** 2022-01-21

**Authors:** Alfonso M. Vidal, Manuel Moya, Sonia Alcalá, Inmaculada Romero, Francisco Espínola

**Affiliations:** 1 Department Chemical, Environmental and Materials Engineering, Universidad de Jaén, Paraje Las Lagunillas, Edif. B-3, 23071 Jaén, Spain; amvidal@ujaen.es (A.M.V.); salcala@ujaen.es (S.A.); iromero@ujaen.es (I.R.); fespino@ujaen.es (F.E.); 2 Center for Advanced Studies in Earth Sciences, Energy and Environment (CEACTEMA), Universidad de Jaén, 23071 Jaén, Spain

**Keywords:** oleuropein, hydroxytyrosol, antioxidant capacity, oxidative stability, kinetic analysis, refined olive oils, phenolic extracts

## Abstract

Refined olive oils (ROOs) are commonly enriched with synthetic antioxidants. Antioxidant extracts obtained from natural products can be used to improve the stability of these oils. In this study, ROOs were enriched through the addition of phenolic extracts from olive leaves (OLs) and exhausted olive pomace (EOP). In addition to replacing synthetic antioxidants with natural ones, this results in the valorization of these olive-derived biomasses. The most suitable method for mixing and enriching refined oils was probe-type ultrasonication using lecithin as the emulsifier. Thereafter, the change in the content of antioxidant compounds and the antioxidant capacity of the oils at 25, 35, and 45 °C were studied over 28 and 50 days of storage. The experimental results were fitted using a pseudo-first-order kinetic model. The oxidative stability index of the ROO enriched with a 2 g/L OL extract (70 h) was higher than that of a commercial ROO (46.8 h). Moreover, the oxidative stability index of the refined olive pomace oil (ROPO) enriched with a 2 g/L EOP extract (44.1 h) was higher than that of a commercial ROPO (38.9 h). In addition, the oxidative stabilities and antioxidant capacities of the oils were significantly correlated.

## 1. Introduction

Olive tree cultivation, virgin olive oil production, and olive oil pomace extraction generate various wastes and byproducts such as leaves, wood, olive pomace, exhausted or defatted olive pomace, and wastewater. Olive trees are primarily cultivated in several countries of the Mediterranean Basin, and millions of tons of these biomasses are produced yearly [1,2]. Sometimes, the generated wastes are difficult to treat, leading to environmental problems. Therefore, the development of alternative uses for these bioresources that can produce high value-added products is a research priority. Recent studies have used the residues and byproducts of olive cultures, such as olive leaves (OLs) [3], olive tree prunings [4], olive pomace [5,6], and exhausted olive pomace (EOP) [7], to obtain biocompounds or energy.

The health benefits of olive oils rich in phenolic compounds have been widely reported [3,8,9,10]. Nevertheless, refined olive oils (ROOs) and refined oils overall lack these compounds because during the refining process of olive oils, secoiridoids are completely lost, while some lignans are saved [11]. Several recent studies have focused on the enrichment of refined oils with phenols [12,13,14,15]. ROOs can be enriched with antioxidants derived from olive byproducts, which are natural sources of phenolic compounds. Tocopherol is typically added to commercial refined oils to increase their stability and prevent their oxidative deterioration [16]. Therefore, researchers have evaluated the nutritional value of enriched olive oils, which are considered functional foods with health benefits [17].

Many methods have been used for extracting antioxidant compounds from biomass residues, including liquid–liquid, microwave- and ultrasound-assisted, and supercritical fluid extraction methods [18,19]. In addition, the recovery of phenolic compounds from these wastes and byproducts contributes to the sustainability of the olive sector and reduces the environmental impact of the wastes and byproducts. These phenolic extracts can be used in the food industry as antioxidants [20].

In this study, the stability and antioxidant properties of ROO and refined olive pomace oil (ROPO) was enhanced. Moreover, the changes in the stabilities and antioxidant properties of OL-extract-enhanced ROOs and EOP-extract-enhanced ROPOs with time were evaluated. The oxidation stability indices (OSIs) of the enriched refined oils were compared with those of commercial oils.

## 2. Materials and Methods

### 2.1. Preparation of OL and EOP Phenolic Extracts

Olive leaves (OLs) cv. Picual (*Olea europaea* L.) were collected from a non-irrigated crop in Cambil (Jaén, Spain). The leaves were oven-dried at 105 °C, ground using a Retsch SM 100 mill (Fisher Scientific SL, Madrid, Spain), and sieved using a 4 mm sieve. Partly stoned and pelletized EOP was obtained from Daniel Espuny, S.A.U., a facility for the industrial extraction of olive pomace oil in Linares-Baeza (Jaén, Spain). The moisture contents of the OL and EOP biomass samples were determined to be 39.1% and 6.7%, respectively, after oven-drying at 105 °C.

The OL phenolic extract was obtained using the procedure described by Lama-Muñoz et al. [21]. In brief, 1 kg of dry OLs was macerated in an ethanol–water solution (80:20 *v*/*v*) under shaking for 24 h at 25 °C. The liquid-to-solid ratio of the mixture was 6:1. Thereafter, the hydroalcoholic extracts were vacuum-filtered to remove the solids and the residual ethanol was evaporated using a rotary evaporator at 40 °C.

The EOP phenolic extract was obtained via hydrothermal treatment in a water bath at 85 °C under shaking for 90 min, using the method of Gómez-Cruz et al. [22]. The liquid-to-solid ratio of the mixture was 10:1. Thereafter, the mixture was filtered to separate the solids from the EOP extract.

Both the OL and EOP extracts were concentrated in an EV-50 vacuum oven (RAYPA, Terrassa, Spain) at 40 °C and stored in amber glasses in a refrigerator at 4 °C to avoid degradation of the phenolic compounds. Once concentrated, OL ethanolic extract was used to enrich the ROO, and EOP aqueous extract was added to the ROPO.

### 2.2. Addition of Extracts to Refined Oils

The process of refining oils involves the loss of their bioactive compounds and therefore of their nutritional and antioxidant values. Commercial refined oils are enriched with synthetic antioxidants to increase their chemical stability. In this context, the use of natural antioxidants from waste biomass is of great value. The refined oils used in this work did not contain additives. They were supplied by the Migasa Group (Seville, Spain) prior to the addition of the synthetic antioxidants. In this study, to improve the antioxidant properties and the stability of the refined oils, the OL and EOP extracts were dissolved by ultrasonication in the ROO and ROPO samples, respectively.

**Figure 1:**
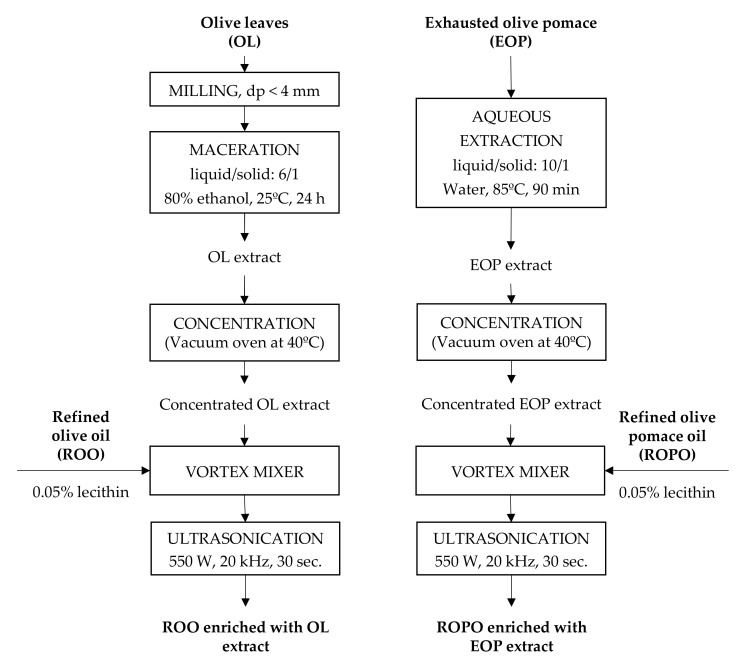
Experimental sequence of phenolic compounds extraction and oil enrichment.

Lecithin was used as an emulsifier to improve mixture stability. L-α-phosphatidylcholine (L-α-lecithin from soybean) was purchased from Sigma-Aldrich (St. Louis, MO, USA). Lecithin doses of 0.05% and 0.1% (*w*/*v*) were used. Lecithin was added to small amounts of ROO and ROPO, and the suspensions were shaken in a vortex agitator. Subsequently, the phenolic extracts were added to the suspensions and the mixtures were shaken (Figure 1).

Ultrasonication was used to homogenize the mixtures of refined oils and phenolic extracts. A probe-type Branson SFX 550 ultrasonic homogenizer (Ultrasonics Corp., Brookfield, CT, USA) was used, operating in the continuous mode at 90% of the maximum power of 550 W and at a frequency of 20 kHz for 30 s. An ice bath was used to prevent the mixtures from heating. The enriched oil samples were stored in amber glasses prior to analysis, and the samples used for the kinetic study were stored at 25, 35, and 45 °C.

### 2.3. Analysis Methods

#### 2.3.1. Extraction Yield

The extraction yields were measured using 2 mL aliquots of the filtered extracts. The liquid samples were evaporated at 105 °C to constant weight. The yields were determined with respect to the amounts of initial dry matter and the results are expressed in grams of extract per gram of dry OL or dry EOP.

#### 2.3.2. Contents of Phenolic Compounds

To identify the phenolic compounds and determine their contents in the extracts and oil samples, the method of Espínola et al. [23] was used with minor modifications. A Model 20 series high-performance liquid chromatograph (Shimadzu, Kyoto, Japan) equipped with a BDS Hypersil C18 column (Thermo Scientific, Waltham, MA, USA) was used. The mobile phase was a ternary gradient made up of Milli-Q water with 0.2% ortho-phosphoric acid, methanol, and acetonitrile. The elution flow rate was 1 mL/min. The oven temperature was set at 30 °C, and the injected volume of sample was 20 μL. The UV detector provided a signal at 280 nm. The phenolic compounds were identified and quantified by comparison with analytical standards of hydroxytyrosol from Extrasynthese (Genay Cedex, France), oleuropein from Fluka (Milan, Italy), and tyrosol, apigenin, apigenin-7-O-glucoside, luteolin-7-O-glucoside, and verbascoside from Sigma-Aldrich (St. Louis, MO, USA). The results are expressed as milligrams of compound per gram of dry extract or per kilogram of oil.

#### 2.3.3. Antioxidant Capacity

The ferric ion reducing antioxidant power (FRAP) values of the samples were determined using colorimetric assays in transparent microplates. The FRAP values were measured using a Bio-Rad iMark microplate absorbance reader (Bio-Rad Laboratories, Hercules, CA, USA) at a wavelength of 595 nm [24]. A Trolox curve was used as the standard, and the results are expressed as the Trolox equivalent (TE) per gram of dry extract.

In addition, a 2,2-diphenyl-1-picrylhydrazyl (DPPH) free radical scavenging assay was used to determine the antioxidant capacities of the samples [25]. The absorbances of the samples were measured at 515 nm using methanol as the blank. The DPPH concentrations were calculated using a calibration curve. An additional calibration curve was used to convert the inhibition percentages into TEs.

#### 2.3.4. Oxidative Stability

The oxidative stabilities of the samples were determined using a Metrohm 679 Rancimat instrument (Metrohm, Herisau, Switzerland). The Rancimat method consists of accelerating the aging of oil samples by increasing their temperature and passing a continuous air stream through them. The air flow carries volatile oxidation products from the sample-containing bottle to a bottle containing distilled water. The conductivity of the water is measured continuously. A significant and sudden increase in conductivity marks the induction time. Various standards describe the Rancimat method [26,27]. A filtered and dry 15 L/h air flow was bubbled into a 3.0 g oil sample contained in a reaction tube at 100 °C. The effluent air containing the volatile organic acids from the sample was collected in a polycarbonate receptacle containing 60 mL of distilled water, and the conductivity of the water was recorded continuously. The OSIs of the samples are expressed in hours.

### 2.4. Kinetic Analysis

To determine the evolution with time and temperature of phenolic compound content and antioxidant capacity, a kinetic study of the refined oils enriched with phenolic extracts was carried out. To the refined olive oil, 2 g of dry OL extract was added per L of oil. To refined olive pomace oil, 2 g of dry EOP extract was added per L of oil. The phenolic compound content and antioxidant capacity were programmed to be analyzed at the beginning of the trials and at 1, 2, 4, 8, 14, 21, and 28 days. In the case of ROPO, as the kinetics could not be easily determined at 28 days, it was necessary to extend the time to 50 days. To study the effect of temperature, the amber vials containing the enriched oils were stored in an oven at 25, 35, and 45 °C. All trials were performed in duplicate, giving a total of 48 amber vials of 50 mL for enriched ROO and another 54 vials for enriched ROPO. The analysis of phenolic compounds was performed individually for the oil in each vial. Antioxidant capacity, FRAP, and DPPH values were determined in triplicate.

The results for the stability and antioxidant properties of the ROO and ROPO samples were processed employing Statgraphics Centurion version XIX, (Statpoint Technologies, Inc., Warrenton, VA, USA). The kinetic data of some samples were fitted using a nonlinear regression and applying the Marquardt algorithm.

### 2.5. Statistical Analysis

An ANOVA analysis with a Bonferroni post hoc test was performed, using Statgraphics Centurion, on the oxidative stability index (OSI) and antioxidant capacity data (FRAP and DPPH methods).

## 3. Results and Discussion

### 3.1. Phenolic Extract Characterization

The extraction yields and antioxidant capacities of the OL and EOP extracts were determined (Table 1). The OL and EOP extracts obtained as described in Section 2.1 were concentrated, adding minimal quantities of water to the oil samples to avoid diluting them. The extraction yields of the OL and EOP samples were determined to be 269.13 and 484.71 g dry extract/kg biomass, respectively. Contreras et al. [24], subjected OLs and EOP to maceration followed by ultrasound-assisted extraction with an ethanol–water solution, and they also reported a higher yield for OL.

**Figure 2:**
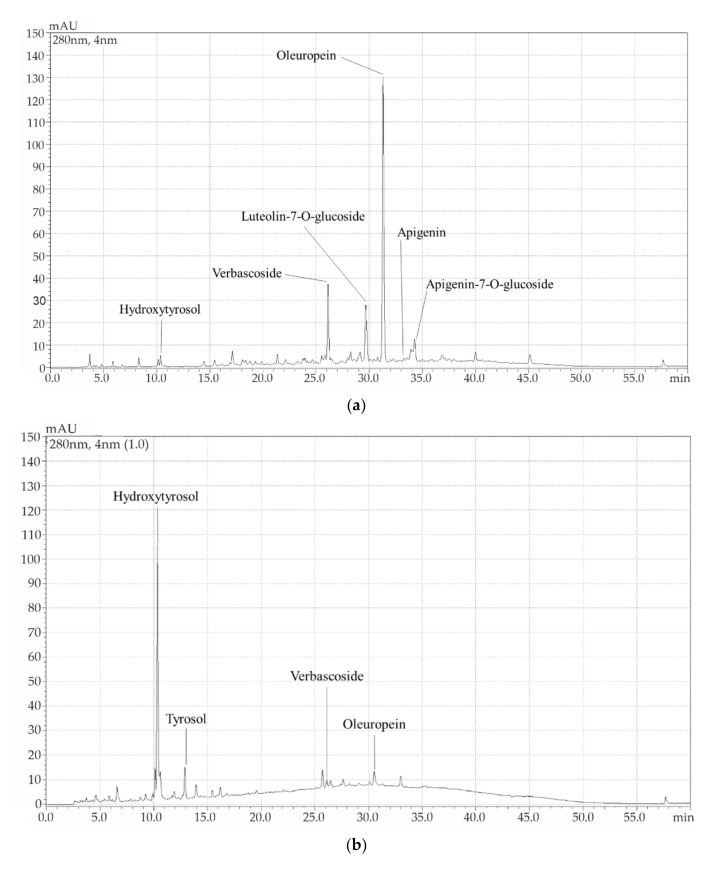
Chromatograms and characterized compounds in the concentrated extract of (**a**) olive leaves and (**b**) exhausted olive pomace.

The OL and EOP extracts were characterized, and their phenolic compositions were determined as indicated in Section 2.3.2. As can be seen in Table 2 and Figure 2a, the main compounds identified in the OL extract were oleuropein, verbascoside, and luteolin-7-O-glucoside. Moreover, the presence of other minor compounds such as hydroxytyrosol, apigenin-7-O-glucoside, and apigenin was also detected. In the EOP extract, hydroxytyrosol was the main phenolic compound identified, together with minor compounds such as tyrosol, oleuropein, and verbascoside (Figure 2b). However, the content of phenolic compounds identified in the OL extract was much higher than in the EOP extract (225.3 versus 19.8 mg/g dry extract). In this study, only the major phenolic compound in each extract was considered, i.e., oleuropein for the OL extract and hydroxytyrosol for the EOP extract.

Although the OL extraction yield was lower than the EOP extraction yield, the antioxidant activities of the OL extract measured using the FRAP and DPPH methods (217.53 and 175.71 mg TE/g extract, respectively) were higher than those of the EOP extract (147.51 and 92.71 mg TE/g extract, respectively) (Table 1). This can be attributed to the higher phenolic compound content in OL extract, mainly oleuropein [18,28]. This agrees with the study by Delgado-Adámez et al. [29], where it was also reported that the contents of phenolic compounds of OL extracts were higher than those of EOP extracts.

### 3.2. Refined Oil Enrichment

Figure 1 shows the experimental procedure used for the enrichment of the refined oils with phenolic extracts. Ultrasonication made the mixtures of the concentrated extracts with the refined oils possible.

Moreover, lecithin was used as the emulsifier to improve the stability of the mixtures of the extracts and the refined oils, as described in Section 2.2. Suárez et al. [14] demonstrated that lecithin addition improved the emulsification of enriched oils. This can be attributed to the amphiphilic behavior of lecithin, which stabilizes the phenolic compounds added to the oil matrices. Other authors used large amounts of lecithin (0.3% *w*/*v*) for emulsification and Polytron homogenizers [12,14]. In the present work, the best results were obtained when the minimum amount of lecithin (0.05% *w*/*v*) was used. Although the amount of lecithin used was small, lecithin served as the emulsifier and stabilizer.

The addition of lecithin to the oil–extract mixtures, followed by vortex agitation and ultrasonication using a probe-type ultrasonic homogenizer achieved stable mixtures. The detailed experimental process was as follows. First, lecithin (0.05% *w*/*v*) was added to a small amount of refined oil, followed by shaking in the vortex agitator. Thereafter, the concentrated phenolic extract was added to the emulsion, and the mixture was shaken. The volume of the added extract was calculated to achieve a concentration of 2 g of dry extract/L of oil. Next, the mixture was added to the remaining refined oil, followed by homogenization using the probe-type ultrasonic homogenizer. Lastly, enriched oil samples with concentrations of 0.5 and 1 g/L were prepared via dilution with refined oil.

### 3.3. Oil Stability

The oxidative stability index and antioxidant capacities of the raw refined oils and the ROO and ROPO samples enriched with different amounts of OL and EOP extracts, respectively, were determined. In addition, the OSIs and antioxidant capacities of commercial refined oils and EVOOs were determined for comparison (Table 3). The addition of the OL extract to ROO caused a noticeable increase in the stability and antioxidant capacity of the ROO, with a positive effect of the extract dose. By contrast, the increase in stability and antioxidant capacity of the EOP-extract-enriched ROPO was limited. The superior OSI and antioxidant capacity of the OL-extract-enriched ROO was attributed to the stronger antioxidant capacity of the OL extract compared with the EOP extract (Table 1). The antioxidant capacity of hydroxytyrosol is higher than those of oleuropein aglycone, tyrosol, and pinoresinol [30]. Nevertheless, considering the primary antioxidant compounds in the extracts used in this study, the oleuropein content of the OL extract was 10 times higher than the hydroxytyrosol content of the EOP extract. Hence, the oxidative stability and useful life of the OL-extract-enriched ROO were higher and longer, respectively, than those of the EOP-extract-enriched ROPO.

**Figure 3:**
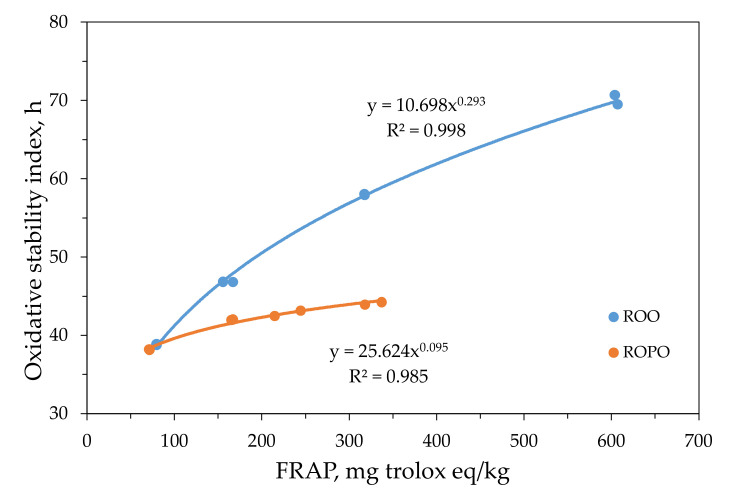
Correlation between oxidative stability index and antioxidant capacity, determined via FRAP, of oil samples: refined olive oil (ROO) and refined olive pomace oil (ROPO).

The OSIs of ROO and ROPO were highly correlated with their antioxidant capacities measured using the FRAP and DPPH methods. The relationship between the OSIs and the antioxidant capacities measured using the FRAP method for ROO and ROPO is illustrated in Figure 3. Franco et al. [31] demonstrated that the oxidative stability of virgin olive oil depended on its antioxidant capacity. Therefore, it can be concluded that these parameters are reliable for estimating the useful life of oils. The oxidative stabilities of the extract-enriched oils equaled or exceeded those of the commercial oils. In particular, the OSI of the ROO enriched with 0.5 g/L of OL extract (ROO-0.5) (45.83 h) was similar to that of a commercial ROO (46.81 h) (Table 3).

The OSIs and antioxidant activities of the two commercial EVOOs were superior to those of the enriched oils in this study.

### 3.4. Kinetic Models

#### 3.4.1. Enriched ROO Models

**Figure 4:**
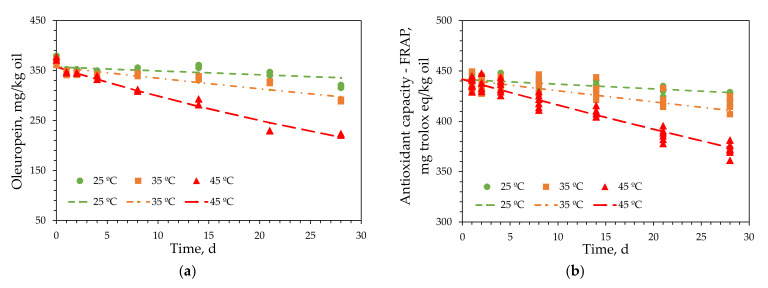
Experimental results for (**a**) oleuropein content and (**b**) antioxidant capacity—FRAP (points) and representation of the kinetic model (lines) for refined olive oil enriched with 2% olive leaves extract.

For the kinetic analysis of the ROO enriched with 2 g/L of OL extract (ROO-2.0), oleuropein was selected as the primary phenolic compound in the oil. The experiments were performed in duplicate, and the results are presented in Figure 4a. The antioxidant capacity was determined in triplicate using the FRAP and DPPH methods, and the results were similar. Therefore, only the results of the FRAP experiments are included herein (Figure 4b).

The experimental results at each temperature were fitted using models that described the degradation kinetics of oleuropein in ROO-2.0 (Figure 4a) and the changes in the antioxidant capacity of ROO-2.0 using the FRAP method (Figure 4b). The integral method was used to determine the kinetic equation, which was derived from the general equation of velocity for a generic reactant A:

(1) $$-\frac{dC_{A}}{dt}=k{\left(C_{A}\right)}^{n}$$

where *C_A_, t, k*, and *n* are concentrations of reactant A, time, kinetic constant, and reaction order, respectively.

As a limit condition, the initial value of *C_A_* was *C_A0_*. As n was unknown, it was assumed that n = 0, 1, and 2, and Equation (1) was integrated for these values of n. The experimental results were adjusted using the obtained equations. The results indicated that the experimental data for the oleuropein kinetics and antioxidant capacities at the three temperatures tested fit the pseudo-first-order reaction model (Equation (2)) the best.

(2) $$C_{A}=C_{A0}e^{-\;kt}$$

Upon substituting *k* in Equation (2) using the Arrhenius equation, Equation (3) was obtained:

(3) $$C_{A}=C_{A0}e^{\left[-\;k_{0}e^{\left(-\;\frac{E_{a}}{RT}\right)}t\right]}\pm \varepsilon$$

where *E_a_* is the activation energy (J/mol), *R* is the gas constant (J/mol K), *T* is the absolute temperature, *k_0_* is the pre-exponential term, and *ε* is the standard deviation of the experimental data from the fitted model. *C_A0_* is independent of the temperature.

This equation was used to determine the kinetic models for the oleuropein content and antioxidant capacity of ROO-2.0.

The parameters in Equation (3) were determined using nonlinear regression and the Levenberg–Marquardt iterative method. Initial parameter values should be provided for this method. Fitting the experimental *C_A_* values with time, *C_A0_* and *k* were obtained at each experimental temperature using Equation (2). *C_A0_* was independent of *T*; therefore, its average value was calculated. Moreover, *k* was determined using the Arrhenius equation. *C_A0_*, *k_0_*, and *E_a_* values were used to solve Equation (3). The kinetic parameters for oleuropein and the antioxidant capacities determined using the FRAP and DPPH methods, together with the corresponding standard deviations, are summarized in Table 4. The standard deviations for all the models used to reproduce the experimental results were low. The coefficients of determination are also included in Table 4, and their values indicate that the experimental data fit the models well. This was confirmed by the plots of the experimental and modeled data shown in Figure 4.

**Figure 5:**
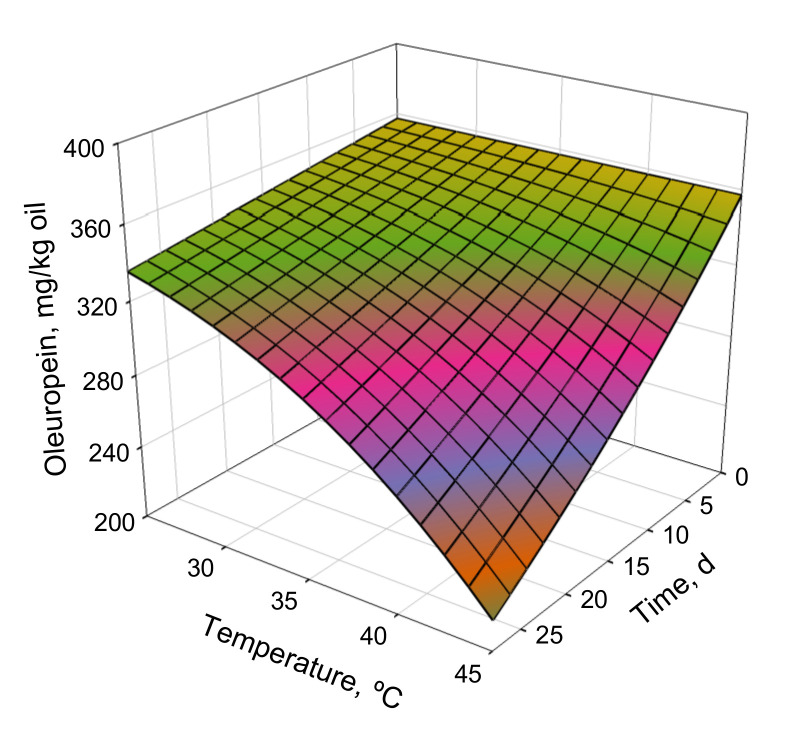
Response surface for oleuropein kinetic model as a function of temperature and time.

The response surface for the oleuropein kinetic model is presented in Figure 3. At low temperatures, the changes in response with time were negligible; however, they increased significantly at higher temperatures. This confirmed that the kinetic process depended significantly on temperature, as suggested by the high *E_a_* values in Table 4. Therefore, the data in Table 4 and Figure 4a and Figure 5 indicate that the reaction kinetics depended significantly on temperature, since the reaction was very slow at low temperatures and increased rapidly with increasing temperature. The response surfaces for the antioxidant capacities, determined using the FRAP and DPPH methods, were similar to that illustrated in Figure 5. Therefore, the similar behaviors of the three responses to temperature indicated that the antioxidant capacities of the enriched olive oils varied proportionally with their oleuropein contents.

The kinetic constants for the three responses of ROO-2.0 are included in Table 4. The low kinetic constants indicated that the reaction rate (Equation (1)) was very low at low temperatures. However, the kinetic constants for oleuropein content and antioxidant capacity increased eight and five times, respectively, upon increasing the temperature from 25 to 45 °C. The *E_a_* values were similar to those reported by Stamatopoulos et al. [32]; however, these authors used higher temperatures (70–100 °C). After the kinetic constants were determined, the times required to decrease the oleuropein content of ROO-2.0 by half at 25, 35, and 45 °C were determined to be 310.89, 106.22, and 38.83 d, respectively. In addition, the time required to decrease the antioxidant capacities determined using the FRAP method by half at 25, 35, and 45 °C were 650.12, 267.58, and 116.45 d, respectively.

Based on the initial oleuropein content of ROO-2.0, its slow degradation kinetics, and the negligible decrease in its antioxidant capacity, it was concluded that the OL-extract-enriched ROO preserved at ≤25 °C can retain its antioxidant properties for at least one year.

#### 3.4.2. Enriched ROPO Models

**Figure 6:**
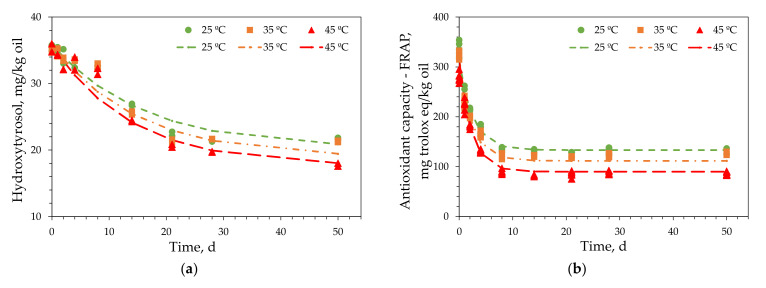
Experimental result (points) for (**a**) hydroxytyrosol and (**b**) antioxidant capacity (FRAP) and representation of the kinetic model (lines) for refined olive pomace oil enriched with 2% exhausted olive pomace extract.

The kinetic analysis of the ROPO enriched with 2 g/L of EOP extract (ROPO-2.0) was also performed using samples stored at 25, 35, and 45 °C. For these experiments, hydroxytyrosol was considered to be the primary phenolic compound in the oil. The experiments for the kinetic study of the hydroxytyrosol content were performed in duplicate, and the results are presented in Figure 6a. Moreover, the antioxidant capacities determined using the FRAP and DPPH methods were determined in triplicate, and the results are illustrated in Figure 6b.

The experiments were initially performed over 28 d, to match the conditions used for the ROO-2.0 analyses. However, the antioxidant capacities asymptotically decreased to a temperature-dependent constant value. Therefore, the experimental time was extended to 50 d and the tests repeated. The results confirmed that the antioxidant capacities remained constant at each temperature. Furthermore, the hydroxytyrosol content, which at 28 d decreased with a tendency to zero, presented an asymptotic trend toward a minimum value after 50 d. These results agree with the findings of Romeo et al. [12], who performed a kinetic study of the enrichment of sunflower oil with olive mill wastewater extract. Therefore, a term that reflects the trend toward a minimum value of the C_A_ response was added to the equation describing the pseudo-first-order kinetic model used to fit the data for ROO (Equation (1)):

(4) $$-\frac{d\left(C_{A}-C_{Am}\right)}{dt}=k\left(C_{A}-C_{Am}\right)$$

As the limit conditions, the initial value of the *C_A_* response is *C_A0_*, which is independent of temperature, whereas the response value at long times is *C_Am_*, which is the minimum value to which the response tended.

By integrating Equation (4) using the aforementioned limit conditions, Equation (5) was obtained:

(5) $$C_{A}=C_{Am}+\left(C_{A0}-C_{Am}\right)e^{-\;kt}$$

The experimental hydroxytyrosol contents and antioxidant capacities determined using the FRAP and DPPH methods at each temperature were determined using Equation (5). The constant term (*C_Am_*) was calculated for each response, and the plot of *C_Am_* as a function of temperature was linear. In addition, upon substituting *k* in Equation (5) using the Arrhenius equation, Equation (6) was obtained. The kinetic data for ROPO-2.0 with respect to time and temperature were determined using Equation (6):

(6) $$C_{A}=\left(aT+b\right)+\left[C_{A0}-\left(aT+b\right)\right]e^{\left[-\;k_{0}e^{\left(-\;\frac{E_{a}}{RT}\right)}t\right]}\pm \varepsilon$$

The initial values of a and b were determined from the plot of *C_Am_* vs. time; moreover, the initial value of the pre-exponential term (*k_0_*) and *E_a_* were obtained using the Arrhenius equation for *k*. These values were used to initiate the nonlinear regression of the experimental results using Equation (6).

The fitted parameters in Equation (6), standard deviations of the model, and coefficients of determination are summarized in Table 5. The model curves of the experimental hydroxytyrosol contents and antioxidant capacities of ROPO-2.0 determined using the FRAP method are presented in Figure 6. The experimental data fit the models well. This was confirmed by the determination coefficients and small standard deviations listed in Table 5.

The data in Table 5 allowed us to determine the changes in the studied responses with time and temperature and revealed the significant dependence of the responses on time at the beginning of the experiments. However, the effect of temperature on the responses was negligible, as indicated by the low *E_a_* values. These results were different from those obtained for ROO-2.0, although increasing the temperature caused the degradation rate of hydroxytyrosol to increase (Figure 6).

**Figure 7:**
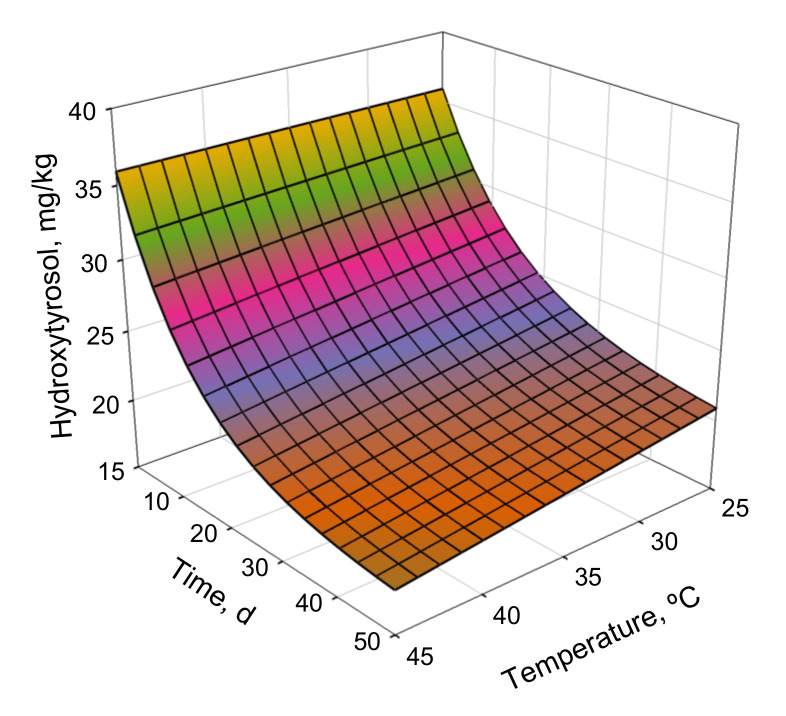
Response surface for hydroxytyrosol kinetic model.

The response surface for the kinetic model used for the hydroxytyrosol content is presented in Figure 7. The asymptotic tendencies toward minimum values at each temperature are also illustrated in Figure 7. Moreover, the plot in Figure 7 illustrates that the main factor that affected the response was time. These findings were supported by the small changes in the kinetic constants with temperature (Table 5). The degradation kinetics of hydroxytyrosol were slow, as indicated by the kinetic constants. Hydroxytyrosol degradation occurred faster than oleuropein degradation; however, the hydroxytyrosol content tended toward a minimum value, which remained constant over time. Moreover, the antioxidant capacity followed a similar trend; it reached a minimum value, which remained constant over time. The minimum achievable values at each temperature are listed in Table 5.

These results indicated that the EOP-extract-enriched ROPO maintained its antioxidant properties over time. Therefore, the added value of these oils, which are primarily used for frying food, can be increased.

## 4. Conclusions

Oleuropein-rich OLs and hydroxytyrosol-rich EOP were used to obtain antioxidant extracts. These extracts were used for enriching ROO and ROPO, respectively.

The antioxidant properties and oxidative stabilities of the enriched refined oils achieved were significantly higher than those of the corresponding commercial refined oils. The addition of 0.5–2% of antioxidant extracts increased the stability of the oils and conferred on them antioxidant properties similar or superior to those of the commercial refined oils used as a reference.

The kinetic study revealed a high correlation between the antioxidant capacities and the oxidative stabilities of the oils. Moreover, the results obtained in this work indicated that hydroxytyrosol degraded faster than oleuropein. The antioxidant extracts obtained from the olive-derived biomass used in this study are promising alternatives to the synthetic antioxidants typically used to enrich ROOs. This study contributes to the valorization of residual olive biomasses in the context of a circular economy model in this sector.

## Figures and Tables

**Table 1:** Extraction yields and antioxidant capacities of OL and EOP concentrated extracts.

	Olive Leaves (OL)	Exhausted Olive Pomace (EOP)
Extraction yield, g dry extract/kg dry biomass	269.13 ± 1.81	484.71 ± 5.20
FRAP, mg Trolox eq/g dry extract	217.53 ± 2.71	147.41 ± 0.66
DPPH, mg Trolox eq/g dry extract	175.71 ± 3.61	92.71 ± 0.39

All values are expressed as mean ± standard deviation.

**Table 2:** Composition of the concentrated extracts in phenolic compounds (expressed as mg compound/g dry extract).

Concentrated Extract	Hydroxytyrosol	Tyrosol	Verbascoside	Luteolin-7-O-Glucoside	Oleuropein	Apigenin	Apigenin-7-O-Glucoside
Olive leaves (OL)	1.68 ± 0.01	-	30.21 ± 0.04	12.85 ± 0.05	180.11 ± 0.04	0.10 ± 0.01	0.34 ± 0.01
Exhausted olive pomace (EOP)	16.69 ± 0.00	2.08 ± 0.01	0.46 ± 0.00	-	0.54 ± 0.00	-	-

All values are expressed as mean ± standard deviation.

**Table 3:** Experimental results for the oxidative stability and antioxidant capacity of ROO enriched with OL extract, ROPO enriched with EOP extract, and commercial oils.

Sample	OSI, h	FRAP, mg Trolox eq/kg oil	DPPH, mg Trolox eq/kg oil
ROO	38.84 ± 0.09 ^a^	79.47 ± 5.41 ^a^	3.21 ± 1.68 ^a^
ROO-0.5	45.83 ± 0.01 ^b^	162.34 ± 8.99 ^b^	79.24 ± 5.22 ^b^
ROO-1.0	58.01 ± 0.11 ^c^	317.51 ± 7.42 ^c^	173.44 ± 5.64 ^c^
ROO-2.0	70.10 ± 0.85 ^d^	605.05 ± 5.05 ^d^	390.93 ± 6.14 ^d^
CROO	46.81 ± 0.64 ^b^	176.11 ± 3.04 ^b^	88.72 ± 4.37 ^b^
ROPO	38.20 ± 0.05 ^a^	71.21 ± 2.87 ^a^	2.80 ± 1.23 ^a^
ROPO-0.5	42.01 ± 0.03 ^a,e^	165.84 ± 1.93 ^b^	15.63 ± 2.03 ^a^
ROPO-1.0	42.83 ± 0.49 ^e^	226.51 ± 18.29 ^e^	48.81 ± 1.85 ^e^
ROPO-2.0	44.08 ± 0.23 ^b,e^	327.41 ± 13.32 ^c^	172.25 ± 8.23 ^c^
CROPO	38.86 ± 0.86 ^a,e^	77.96 ± 3.64 ^a^	4.23 ± 1.89 ^a^
EVOO-A	84.56 ± 1.58 ^f^	705.19 ± 14.76 ^f^	368.94 ± 7.81 ^f^
EVOO-B	77.75 ± 0.81 ^g^	649.18 ± 15.06 ^g^	337.75 ± 6.58 ^g^

All values are expressed as mean ± standard deviation. In each column, different letters (superscripts) indicate significant differences between the data (*p* < 0.05). ROO: refined olive oil; ROO-0.5: ROO enriched with 0.5% (*v*/*v*) phenolic extract; ROO-1.0: ROO enriched with 1% of phenolic extract; ROO-2.0: ROO enriched with 2% of phenolic extract; CROO: commercial ROO; ROPO: refined olive pomace oil; ROPO-0.5: ROPO enriched with 0.5% of phenolic extract; ROPO-1.0: ROPO enriched with 1% of phenolic extract; ROPO-2.0: ROPO enriched with 2% of phenolic extract; CROPO: commercial ROPO; EVOO: extra virgin olive oil, A and B.

**Table 4:** Kinetic parameters of Equation (3), standard deviation (*ε*), and coefficient of determination (R^2^), for oleuropein content (mg/kg) and antioxidant capacity (mg Trolox eq/kg) and calculated kinetic constants at operating temperatures.

		Antioxidant Capacity	
	Oleuropein	FRAP	DPPH
*C_A0_*	357.10	441.61	257.69
*k_0_*, d^−1^	5.2519·10^11^	8.0972·10^8^	1.7152·10^8^
*E_a_*, kJ/mol	82.036	67.814	61.556
ε	8.81	5.34	5.87
R^2^	0.951	0.918	0.933
Temperature, °C	*k*, d^−1^		
25	0.0022	0.0011	0.0028
35	0.0065	0.0026	0.0063
45	0.0179	0.0060	0.0134

**Table 5:** Kinetic parameters of Equation (6), standard deviation (*ε*), and coefficient of determination (R^2^) for hydroxytyrosol content (mg/kg) and antioxidant capacity (mg Trolox eq/kg), and calculated values for *k* and *C_Am_* at operating temperatures.

		Antioxidant Capacity	
	Hydroxytyrosol	FRAP	DPPH
*C_A0_*	35.91	312.25	165.54
*a*	−0.1327	−2.1673	−1.0804
*b*	59.771	779.44	405.45
*k_0_*, d^−1^	0.6708	2.3784	1897.78
*E_a_*, kJ/mol	5.862	4.436	20.179
*ε*	1.24	8.74	3.55
R^2^	0.964	0.981	0.988
Temperature, °C	*K,* d^−1^		
25	0.0630	0.3973	0.5534
35	0.0681	0.4211	0.7207
45	0.0731	0.4446	0.9231
Temperature, °C	*C_Am_*		
25	20.21	133.28	83.32
35	18.88	111.60	72.52
45	17.55	89.93	61.71

## Data Availability

Data are contained within the article.

## Abbreviations

CROO	commercial refined olive oil
CROPO	commercial refined olive pomace oil
DPPH	2,2-diphenyl-1-picrylhydrazyl
EOP	exhausted olive pomace
EVOO	extra virgin olive oil
FRAP	ferric ion reducing antioxidant power
OL	olive leaf
OSI	oxidative stability index
ROO	refined olive oil
ROPO	refined olive pomace oil
TE	Trolox equivalent
